# The incidence, risk factors and maternal and foetal outcomes of uterine rupture during different birth policy periods: an observational study in China

**DOI:** 10.1186/s12884-021-03811-8

**Published:** 2021-05-05

**Authors:** Yangwen Zhou, Yi Mu, Peiran Chen, Yanxia Xie, Jun Zhu, Juan Liang

**Affiliations:** 1 National Office for Maternal and Child Health Surveillance of China, West China Second University Hospital, Sichuan University, No. 17 Ren Min Nan Lu, Chengdu City, Sichuan Province 610041 P. R. China; 2 National Office for Maternal and Child Health Surveillance of China, Department of Obstetrics, West China Second University Hospital, Sichuan University, Chengdu, Sichuan China; 3 Key Laboratory of Birth Defects And Related Diseases of Women and Children (Sichuan University), Ministry of Education, Chengdu, Sichuan China; 4 National Center for Birth Defect Monitoring of China, West China Second University Hospital, Sichuan University, No. 17 Ren Min Nan Lu, Chengdu City, Sichuan Province 610041 P. R. China

**Keywords:** Uterine rupture, Birth policy, Incidence, Risk factors, Pregnancy outcomes

## Abstract

**Background:**

Currently, there are no studies on changes in the incidence of uterine rupture or maternal and foetal outcomes in women with uterine rupture during different birth policy periods in China. Moreover, the results of association studies of maternal age, parity and previous caesarean section number with the risk of maternal and foetal outcomes in women with uterine rupture have not been consistent. This research aims to conduct and discuss the above two aspects.

**Methods:**

We included singleton pregnant women with no maternal complications other than uterine rupture from January 2012 to June 2019 in China’s National Maternal Near Miss Surveillance System. The data in this study did not differentiate between complete and partial uterine rupture and uterine dehiscence. Through Poisson regression analysis with a robust variance estimator, we compared the incidences of uterine rupture and maternal and foetal outcomes in women with uterine rupture during different birth policy periods in China and determined the relationship between maternal age, parity or previous caesarean section number and uterine rupture or maternal and foetal outcomes in women with uterine rupture.

**Results:**

This study included 8,637,723 pregnant women. The total incidences of uterine rupture were 0.13% (12,934) overall, 0.05% during the one-child policy, 0.12% during the partial two-child policy (aRR = 1.96; 95% CI: 1.53–2.52) and 0.22% (aRR = 2.89; 95% CI: 1.94 4.29) during the universal two-child policy. The maternal near miss and stillbirth rates in women with uterine rupture were respectively 2.35% (aRR = 17.90; 95% CI: 11.81–27.13) and 2.12% (aRR = 4.10; 95% CI: 3.19 5.26) overall, 5.46 and 8.18% during the first policy, 1.72% (aRR = 0.60; 95% CI: 0.32–1.17) and 2.02% (aRR = 0.57; 95% CI: 0.37–0.83) during the second policy, and 1.99% (aRR = 0.90; 95% CI: 0.52–1.53) and 1.04% (aRR = 0.36; 95% CI: 0.24–0.54) during the third policy. The risk of uterine rupture increased with parity and previous caesarean section number.

**Conclusion:**

The uterine rupture rate in China continues to increase among different birth policy periods, and the risk of maternal near miss among women with uterine rupture has not significantly improved. The Chinese government, obstetricians, and scholars should work together to reverse the rising rate of uterine rupture and improve the pregnancy outcomes in women with uterine rupture.

**Supplementary Information:**

The online version contains supplementary material available at 10.1186/s12884-021-03811-8.

## Background

Uterine rupture is an emergency obstetric condition in which the uterine wall is torn and the integrity of the uterus is destroyed, often causing serious maternal and perinatal complications [1–3]. The incidence of uterine rupture is low. According to a systematic review by the World Health Organization (WHO), the incidence of uterine rupture based on community research is 0.053% and that based on medical institution research is 0.31% [4].

In November 2013 and October 2015, China implemented the partial two-child policy [5] and the universal two-child policy [6], respectively. The numbers and proportions of older women, multiparous women and women with previous caesarean sections have been constantly increasing [7, 8], which may lead to maternal and neonatal health problems. It has been reported that compared with those during the period of the one-child policy, the incidences of maternal placental accreta spectrum disorder [9] and birth defects [10] during the period of the universal two-child policy have increased. Uterine rupture is an obstetric disease that is closely related to maternal reproductive characteristics. The changes in these reproductive characteristics may also affect the uterine rupture incidence and maternal and neonatal outcomes. To our knowledge, there is no research report on the incidence of uterine rupture or the changes in maternal and foetal outcomes in women with uterine rupture under the different birth policies.

Previous caesarean section delivery is the most important risk factor for uterine rupture [11]. However, whether the risk of uterine rupture increases with the increasing number of previous caesarean sections is still unclear [12, 13]. Moreover, according to the 2019 American College of Obstetricians and Gynecologists (ACOG) guidelines for vaginal birth after caesarean section delivery [14], women with two previous low-transverse caesarean deliveries are candidates for trial of labour after caesarean delivery (TOLAC) [14], but the expert consensus on the management of vaginal birth after caesarean section delivery in China (2016) indicates that a history of two uterine operations is a contraindication for TOLAC [15]. At the same time, the ACOG guideline [14] and Chinese expert consensus [15] do not mention the influence of maternal age or parity on uterine rupture, and there are also contradictions among research study results [12, 13, 16–20]. These findings suggest that the relationship between the risk of uterine rupture and maternal age, parity, or the number of previous caesarean sections needs more research evidence.

The purpose of this study was to identify the trends in uterine rupture among different birth policy periods and changes in maternal and foetal outcomes in women with uterine rupture in China, to analyse the relationship between maternal age, parity or number of previous caesarean sections and uterine rupture or maternal and foetal outcomes and to provide basic data to inform obstetric consulting and the optimization of obstetric management and practice.

## Methods

### Data sources

We used data collected from January 2012 to June 2019 from China’s National Maternal Near Miss Surveillance System (NMNMSS). The surveillance system, which was established in October 2010, covered 326 urban or rural areas in 30 provinces and covered 441 hospitals with more than 1000 annual births at the county level or above. We used the maternal and perinatal health survey method [21] recommended by the WHO to record the information regarding maternal near misses and foetal outcomes in monitoring hospitals. The obstetrician prospectively collected data on the maternal sociodemographic and obstetric characteristics of all pregnant or postpartum women admitted to the obstetric department at each monitoring hospital from the time of admission until discharge. The sampling strategy, data collection, reporting processes, and quality control method of the monitoring system have been described in previous studies [22, 23].

This study was approved by the Ethics Review Committee of West China Second University Hospital, Sichuan University, and conducted in accordance with the principles of the Declaration of Helsinki. Because of the retrospective design of this study, the Ethics Review Committee of West China Second University Hospital, Sichuan University has waived the requirement of the informed consent for this study.

### Definition of variables

Given the time interval between conception and delivery, the effect of the new policy will lag. We delayed the time of implementation of the partial two-child policy and the universal two-child policy by 9 months to use the actual starting times of the effects of the two birth policies, namely, October 2014 and September 2016, respectively. Based on this, the period of the one-child policy in this study was from January 2012 to August 2014 (31 months), the period of the partial two-child policy was from September 2014 to July 2016 (22 months), and the period of the universal two-child policy was from August 2016 to June 2019 (34 months).

We divided maternal age into the following groups: < 24, 24–29, 30–34 and ≥ 35 years. Parity did not include this pregnancy and was classified into 0, 1, 2 and ≥ 3, and the number of previous caesarean sections was divided into 0, 1 and ≥ 2. Uterine rupture was defined as uterine or lower uterine dehiscence in late pregnancy or during childbirth, including complete and incomplete rupture [24], and cases of uterine dehiscence found during elective caesarean section without preceding clinical symptoms were also included in the study. The adverse pregnancy outcomes focused on in this study were maternal near miss and fetal stillbirth. The criteria for maternal near miss were based on the organ failure criteria recommended by the WHO [25]. Stillbirth included antepartum and intrapartum fetal death. Other variables included region (eastern, central and western), hospital level (unknown, level 1, level 2, or level 3), maternal education (no school, primary school, middle school, high school, college or higher), marital status (married, single/widowed/divorced), number of antenatal care visits (0, 1–3, 4–6, 7–9, 10 or higher), gestational age (< 28 weeks, 28–31 weeks, 32–36 weeks, 37–42 weeks, or 42 weeks or higher), foetal presentation (head or other form of presentation), delivery method (vaginal delivery or caesarean section delivery), foetal sex (male or female), and foetal weight (< 2500 g, 2500–4000 g, or > 4000 g). The foetal delivery method was the final delivery method.

### Statistical analysis

The study used data from 438 of 441 monitoring hospitals (excluding three medical institutions that had not reported any data since 2012), restricted the analysis to women who delivered at or after 28 completed weeks of gestation and excluded women with pregnancy complications other than uterine rupture.

First, we estimated the total incidence of uterine rupture in China from 2012 to 2019 and the incidences of uterine rupture in China during the different birth policy periods. As the NMNMSS oversampled hospitals in large cities, we weighted the uterine rupture rate for the sampling distribution of the population according to the 2010 census of China, as detailed elsewhere [22]. To examine the relationships between different birth policy periods and uterine rupture rates, we used Poisson regression with a robust variance estimator [26, 27]. We calculated crude relative risks (cRRs) and 95% CIs after weighting for the sampling distribution of the population and clustering of births within hospitals. We also calculated adjusted relative risks (aRRs) and 95% confidence intervals (CIs) by further adjusting for the hospital (region and hospital level), maternal sociodemographic characteristics (education level, marital status, and number of antenatal care visits), and maternal obstetric characteristics (foetal presentation, delivery methods, foetal sex, and foetal weight). We investigated both multicollinearity and model goodness-of-fit to identify the most robust and stable model.

Then, we used the above methods to determine the association between maternal age, parity of the number of previous caesarean deliveries and uterine rupture to estimate the rates of maternal near miss and stillbirth in women with uterine rupture under different birth policy periods, to compare the risks of adverse pregnancy outcomes between women with uterine rupture and women without uterine rupture, to compare the risks of adverse pregnancy outcomes in women with uterine rupture during the different birth policy periods, to examine the relationship between maternal age, parity or the number of previous caesarean deliveries and adverse pregnancy outcomes in women with uterine rupture and outcomes among women with uterine rupture, computing their cRRs, aRRs, and 95% CIs. All the above analyses were completed with STATA 15.0.

## Results

### Changes in the uterine rupture rate in China under different birth policy periods

From January 2012 to June 2019, a total of 12,934 cases of uterine rupture occurred among 8,637,723 singleton pregnant women, with a rupture rate of 0.13% (Table 2). Compared with that during the period of the one-child policy, which was 0.05%, the rate of uterine rupture increased by 0.12% (aRR = 1.96; 95% CI: 1.53–2.52) and 0.22% (aRR = 2.89; 95% CI: 1.94–4.29) during the periods of the partial two-child policy and universal two-child policy. This trend was also observed among women of different ages, parities and numbers of previous caesarean sections (Table 1). During the period of the universal two-child policy, the uterine rupture rates of pregnant women aged 35 and above, of those who had one or more deliveries, and of those who had one or more previous caesarean sections were 0.42% (aRR = 2.80; 95% CI: 1.72–4.58), 0.39% (aRR = 3.05; 95% CI: 1.99–4.65) and 1.02% (aRR = 3.21; 95% CI: 2.06–4.98), respectively (Table 1).

**Table 1:** Incidence and number of uterine ruptures by maternal age, parity and number previous caesarean sections over different birth policy periods in 438 hospitals, China

Maternal characteristics	One-child policy period	Partial two-child policy period	Universal two-child policy period
**Weighted rate, per 100 births**^**a**^			
**Maternal age**			
**<** 24	0.02	0.05	0.09
24–29	0.04	0.09	0.16
30–34	0.08	0.21	0.32
≥ 35	0.65	0.24	0.42
Missing	0.04	0.09	0.10
**Parity**			
0	0.01	0.02	0.02
**≥ 1**			
All	0.10	0.23	0.39
1	0.09	0.23	0.38
2	0.13	0.26	0.46
≥ 3	0.14	0.15	0.28
Missing	0.03	0.00	1.21
**Previous caesarean sections**			
0	0.01	0.02	0.02
≥ 1			
All	0.30	0.67	1.02
1	0.28	0.64	0.95
≥ 2	0.65	1.18	1.92
Missing	0.02	0.14	1.09
**Total**	0.05	0.12	0.22
**Adjusted odds ratio**^**a**^			
**Maternal age**^**b**^			
**<** 24	1	**1.79 (1.31–2.45)**	**2.54 (1.75–3.68)**
24–29	1	**1.86 (1.40–2.44)**	**2.90 (1.94–4.35)**
30–34	1	**2.00 (1.49–2.69)**	**2.84 (1.82–4.32)**
≥ 35	1	**2.06 (1.47–2.87)**	**2.80 (1.72–4.58)**
**Parity**^**c**^			
0	1	**1.50 (1.12–1.99)**	**1.89 (1.24–2.88)**
≥ 1			
All	1	**2.05 (1.56–2.70)**	**3.05 (1.99–4.65)**
1	1	**2.09 (1.57–2.77)**	**3.00 (1.92–4.70)**
2	1	**1.74 (1.31–2.31)**	**2.75 (1.86–4.06)**
≥ 3	1	0.97 (0.56–1.69)	**1.83 (1.09–3.06)**
**Previous caesarean sections**^**d**^			
0	1	**1.45 (1.14–1.85)**	**1.88 (1.37–2.59)**
≥ 1			
All	1	**2.14 (1.60–2.86)**	**3.21 (2.06–4.98)**
1	1	**2.12 (1.57–2.87)**	**3.06 (1.93–4.85)**
≥ 2	1	**1.82 (1.36–2.44)**	**3.20 (2.12–4.84)**
**Total**	1	**1.96 (1.53–2.52)**	**2.89 (1.94–4.29)**

^a^Weighted for the sampling distribution of the population covered by the Chinese National Maternal Near Miss Surveillance System

^b^We did not adjust for maternal age in the different age groups

^c^We did not adjust for parity in the different parity groups

^d^We did not adjust for previous caesarean sections in different groups of previous caesarean sections

### Relationships between uterine rupture and maternal age, parity and number of previous caesarean sections

Multiparity (aRR = 2.17; 95% CI: 1.43–3.29) and previous caesarean section history (aRR = 11.33; 95% CI: 8.59–14.96) were risk factors for uterine rupture, and the risk of uterine rupture increased with increasing parity and number of previous caesarean sections (Table 2). The risk of uterine rupture for women with 1, 2 and 3 or more parity was respectively 2.21-fold, 2.60-fold and 2.57-fold that of women with 0 parity (Table 2). The risk of uterine rupture for women with 1 and 2 or more previous caesarean sections was 10.69 times and 19.93 times that of women with 0 caesarean sections (Table 2).

**Table 2:** The risk of uterine rupture by age, parity and number of previous caesarean sections in China

Maternal characteristics	Number of uterine ruptures (%)	Weighted rate, per 100 births^**a**^	Crude odds ratio^**a**^	Adjusted odds ratio^**a**^
**Maternal age**^**b**^				
**<** 24	1105 (8.54)	0.05	1	1
24–29	3949 (30.53)	0.10	**2.11 (1.71–2.62)**	0.91 (0.78–1.07)
30–34	4830 (37.34)	0.23	**4.67 (3.45–6.34)**	0.91 (0.72–1.14)
≥ 35	2831 (21.89)	0.29	**6.05 (4.02–9.10)**	0.83 (0.66–1.04)
Missing	219 (1.69)	0.07	1.40 (0.94–2.10)	**0.55 (0.38–0.79)**
**Parity**^**c**^				
0	948 (7.33)	0.02	1	1
≥ 1				
All	–	0.26	**14.69 (9.31–23.20)**	**2.17 (1.43–3.29)**
1	9698 (74.98)	0.26	**14.21 (8.85–22.81)**	**2.21 (1.45–3.37)**
2	1966 (15.20)	0.32	**17.74 (11.55–27.26)**	**2.60 (1.68–4.02)**
≥ 3	225 (1.74)	0.21	**11.46 (7.28–18.04)**	**2.57 (1.66–3.99)**
Missing	97 (0.75)	0.91	**50.92 (25.49–101.72)**	2.79 (0.91–8.57)
**Previous caesarean sections**^**d**^				
0	1545 (11.95)	0.02	1	1
≥ 1				
All	–	0.75	**39.85 (26.99–58.84)**	**11.33 (8.59–14.96)**
1	9797 (75.75)	0.70	**37.42 (25.04–55.94)**	**10.69 (8.15–14.02)**
≥ 2	1478 (11.43)	1.50	**79.52 (53.76–117.62)**	**19.93 (13.16–30.20)**
Missing	114 (0.88)	0.29	**15.38 (2.94–80.42)**	1.29 (0.34–4.87)
**Total**	12,934 (100.00)	0.13	**–**	–

^a^Weighted for the sampling distribution of the population covered by the Chinese National Maternal Near Miss Surveillance System

^b^We did not adjust for maternal age in the different age groups

^c^We did not adjust for parity in the different parity groups

^d^We did not adjust for previous caesarean sections in different groups of previous caesarean sections

### Relationships between adverse maternal and foetal outcomes of women with uterine rupture and maternal age, parity and number of previous caesarean sections

The rates of maternal near miss and stillbirth among women with uterine rupture were 2.35 and 2.12%, respectively, and the risks were 17.90-fold (aRR = 17.90,95% CI 95% CI: 11.81–27.13) and 4.10-fold (aRR = 4.10,95% CI 95% CI: 3.19–5.26) that among women without uterine rupture (Table 3).

**Table 3:** Adverse maternal and foetal outcomes by age, parity and number of previous caesarean sections among women in China

	Maternal near miss				Stillbirth			
	Number (%)	Weighted rate, per 100 births^**a**^	Crude odds ratio^**a**^	Adjusted odds ratio^**a,b**^	Number (%)	Weighted rate, per 100 births^**a**^	Crude odds ratio^**a**^	Adjusted odds ratio^**a,b**^
**Without uterine rupture**	5130 (94.87)	0.05	**1**	**1**	92,413 (99.73)	1.01	**1**	**1**
**With uterine rupture**	276 (5.13)	2.35	**48.12 (33.44–69.24)**	**17.90 (11.81–27.13)**	250 (0.27)	2.12	**2.10 (1.40–3.15)**	**4.10 (3.19–5.26)**
**With uterine rupture**								
**Maternal age**^**c**^								
**<** 24	28 (10.14)	2.74	1	**1**	37 (14.80)	3.27	1	**1**
24–29	74 (26.81)	2.06	0.75 (0.47–1.19)	0.87 (0.52–1.46)	77 (30.80)	2.15	0.66 (0.42–1.03)	1.19 (0.76–1.87)
30–34	90 (32.61)	2.00	0.73 (0.43–1.22)	0.84 (0.49–1.46)	73 (29.20)	1.70	**0.52 (0.31–0.85)**	1.24 (0.79–1.95)
≥ 35	71 (25.72)	2.78	1.01 (0.61–1.70)	1.00 (0.57–1.72)	50 (20.00)	1.83	0.56 (0.31–1.01)	1.09 (0.65–1.81)
Missing	13 (4.71)	8.34	3.05 (1.47–6.30)	1.64 (0.74–3.62)	13 (5.20)	8.98	**2.74 (1.33–5.67)**	**2.61 (1.27–5.34)**
**Parity**^**d**^								
0	43 (15.58)	4.56	**1**	**1**	43 (17.20)	4.66	1	**1**
1	151 (54.71)	1.69	0.37 (0.22–0.63)	1.42 (0.75–2.68)	135 (54.00)	1.47	**0.32 (0.17–0.60)**	1.09 (0.58–2.05)
2	63 (22.83)	3.82	0.84 (0.50–1.41)	**3.31 (1.62–6.77)**	54 (21.60)	3.53	0.76 (0.40–1.44)	**2.15 (1.09–4.27)**
≥ 3	19 (6.88)	10.71	**2.35 (1.25–4.41)**	**4.73 (2.04–10.93)**	18 (7.20)	9.36	2.01 (0.88–4.60)	**3.21 (1.54–6.65)**
Missing	0 (0.00)	0	**–**	**–**	0 (0.00)	0	–	**–**
**Previous caesarean sections**^**e**^								
0	95 (34.42)	6.77	**2.74 (1.63–4.58)**	**4.68 (2.50–8.76)**	92 (36.80)	6.42	**2.84 (1.61–4.99)**	**4.47 (2.58–7.74)**
1	147 (53.26)	1.64	0.66 (0.41–1.09)	**2.18 (1.30–3.65)**	127 (50.80)	1.46	0.64 (0.40–1.02)	**2.62 (1.54–4.56)**
≥ 2	32 (11.59)	2.48	**1**	**1**	31 (12.40)	2.26	**1**	**1**
Missing	2 (0.72)	1.63	0.66 (0.07–5.98)	9.17 (1.23–67.55)	0 (0.00)	0	–	**–**

^a^Weighted for the sampling distribution of the population covered by the Chinese National Maternal Near Miss Surveillance System

^b^Adjusted for the clustering of births within hospitals; region; hospital level; maternal sociodemographic characteristics (maternal age, parity, previous caesarean section, education level, marital status, and number of antenatal care visits); and maternal obstetric characteristics (foetal presentation, delivery methods, foetal sex, and foetal weight)

^c^We did not adjust for maternal age in the different age groups

^d^We did not adjust for parity in the different parity groups

^e^We did not adjust for previous caesarean sections in different groups of previous caesarean section

The risks of maternal near miss and stillbirth increased with increasing parity among women with uterine rupture. Additionally, the risks of maternal near miss among women with a parity of 2 and 3 or more were respectively 3.31-fold (aRR = 3.31, 95% CI: 1.62–6.77) and 4.73-fold (aRR = 4.73, 95% CI: 2.04–4.27) those of women with a parity of 0, and the risks of stillbirth among women with a parity or 2 and 3 or more were respectively 2.15-fold (aRR = 2.15, 95% CI: 1.09–4.27) and 3.21-fold (aRR = 3.21, 95%CI: 1.54–6.65) those of women with a parity of 0, respectively (Table 3).

As the number of previous caesarean sections decreased, the risks of maternal near miss and stillbirth increased among women with uterine rupture. The risks of maternal near miss among women with a parity of 1 and parity of 0 were respectively 2.18-fold (aRR = 2.18, 95%CI: 1.30–3.65) and 4.68-fold (aRR = 4.68, 95% CI: 2.50–8.76) those of women with a parity of 2, and the risks of stillbirth were respectively 2.62-fold (aRR = 2.62, 95% CI: 1.54–4.56) and 4.47-fold (aRR = 4.47, 95% CI: 2.58–7.74) those of women with a parity of 2 (Table 3). After multiple-factor adjustment, there was no statistically significant difference between maternal age and the risk of maternal near miss or stillbirth among women with uterine rupture (Table 3).

### Changes in adverse maternal and foetal outcomes in women with uterine rupture in China during different birth policy periods

In this study, the rates of maternal near miss and stillbirth in women with uterine rupture were 2.35 and 2.12%, respectively (Table 3). The risks of maternal near miss (aRR = 17.90, 95% CI: 11.81–27.13) and stillbirth (aRR = 4.10, 95% CI: 3.19–5.26) were increased by uterine rupture (Table 4). The stillbirth rates in women with uterine rupture were 2.02 and 1.04% during the periods of the partial two-child policy and universal two-child policy, respectively, and 8.18% during the period of the one-child policy, and the stillbirth risks decreased by 43% (aRR = 0.57, 95% CI: 0.37–0.83) and 64% (aRR = 0.36, 95% CI: 0.24–0.54), respectively (Table 4). There were differences in the maternal near miss rates in women with uterine rupture among the different birth policy periods, but there was no statistically significant difference in the risk after multiple-factor adjustment (Table 4).

**Table 4:** The risks of maternal near miss and stillbirth in women with uterine rupture during different birth policy periods

Maternal characteristics	One-child policy period	Partial two-child policy period	Universal two-child policy period
**Number (%)**			
Maternal near miss	75 (27.7)	45 (16.30)	156 (56.52)
Stillbirth	105 (42.00)	60 (24.00)	85 (34.00)
**Weighted rate, per 100 births**^**a**^			
Maternal near miss	5.46	1.72	1.99
Stillbirth	8.18	2.02	1.04
**Crude odds ratio**^**a**^			
Maternal near miss	1	**0.32 (0.19–0.52)**	**0.36 (0.21–0.65)**
Stillbirth	1	**0.25 (0.16–0.38)**	**0.13 (0.08–0.20)**
**Adjusted odds ratio**^**a,b**^			
Maternal near miss	1	0.60 (0.32–1.17)	0.90 (0.52–1.53)
Stillbirth	1	**0.57 (0.37–0.83)**	**0.36 (0.24–0.54)**

^a^Weighted for the sampling distribution of the population covered by the Chinese National Maternal Near Miss Surveillance System

^b^Adjusted for the clustering of births within hospitals; region; hospital level; maternal sociodemographic characteristics (maternal age, parity, previous caesarean sections, education level, marital status, and number of antenatal care visits); and maternal obstetric characteristics (foetal presentation, delivery methods, foetal sex, and foetal weight)

In Additional file 1, we present number of uterine ruptures by maternal age, parity and number previous caesarean sections over different birth policy periods in 438 hospitals in China. In Additional file 2, we present the changes in maternal characteristics during the different birth policy periods in this study. In Additional file 3, we present the influence of parity and the number of previous caesarean sections on the incidence of uterine rupture after stratification by age. Interested readers can go to the relevant webpage to download and reference this information.

## Discussion

We analysed data on 8,637,723 pregnancies from 438 monitoring hospitals in China from January 2012 to June 2019. From the period of the one-child policy to the partial two-child policy and universal two-child policy, the uterine rupture rate increased, and the stillbirth risk among women with uterine rupture decreased; furthermore, no statistically significant difference was observed the risk of maternal near miss among women with uterine rupture. The risk of uterine rupture increased with parity and the number of previous caesarean sections. The higher the parity was, the lower the number of previous caesarean sections and more serious the maternal and foetal outcomes in women with uterine rupture would be. The risks of maternal near miss and stillbirth among women with uterine rupture were significantly higher than those among women without uterine rupture.

During the periods of the one-child policy, partial two-child policy and universal two-child policy, the rates of uterine rupture in China increased from 0.05 to 0.12% to 0.22%, respectively. Studies conducted in Israel [2] from 1988 to 2009 and Norway [28] from 1967 to 2008 also found that the rate of uterine rupture was constantly increasing. In this study, we estimated the uterine rupture rate in China was 0.13%, which is lower than the hospital-based median rate 0.31% reported in the WHO systematic review [4]. The reason may be that the literature sources in this systematic review were mainly from countries in Africa, Asia, Latin America and the Middle East, leading to a higher uterine rupture rate. The uterine rupture rate in China is higher than that reported by the International Network of Obstetric Survey Systems (INOSS) [29], the Nordic obstetric monitoring study [30] and the United States [31]. One of the main reasons may be that the complete uterine rupture rate was only estimated in those articles, while the uterine rupture in our study included complete uterine rupture, partial uterine rupture and asymptomatic dehiscence.

Consistent with previous studies, this study found that previous caesarean section was a risk factor for uterine rupture [1, 32, 33]. Moreover, the risk of uterine rupture increases with increasing number of previous caesarean sections, which was consistent with the British study [13] and contradicted by the WHO study [12]. Moreover, previous studies suggested that the rapid increase in the uterine rupture rate in the past few decades was closely related to the increase in the caesarean section rate [2, 28, 33, 34]. Reducing the rate of nonmedical indications for caesarean sections among pregnant women, especially nulliparous women, remains a priority in reducing the global rate of uterine rupture. In this study, older age was not a risk factor for uterine rupture, which was consistent with the results of the two studies [12, 13]; however, studies from the Netherlands [18], Sweden [16] and Norway [17, 19] showed that age over 35 or 40 years significantly increases the risk of uterine rupture. We found that parity was a risk factor for uterine rupture, which was consistent with the results of the studies from Norway [19] and Denmark [20] and inconsistent with other studies [13, 17, 18].

In the study, the maternal near miss and stillbirth rates in women with uterine rupture were 2.35 and 2.12%, respectively. According to the WHO [12], the maternal near miss and stillbirth rates in women with uterine rupture with a history of caesarean section were 31.2 and 42.9%, respectively, which were significantly higher than the rates in our study. In the Dutch [18] and British [13] studies, the perinatal mortality rates among women with uterine rupture were 8.70 and 12.4%, respectively, which were also higher than the rates in our study. On the one hand, perinatal mortality in Dutch and British studies included neonatal deaths, and this study only inlcuded stillbirth. On the other hand, incomplete rupture and cases of uterine dehiscence were also included in this study, which was different with in both studies. The stillbirth rate among women with uterine rupture dropped from 8.18 to 1.04% over the three periods of the child policy, and the risk of stillbirth dropped to 64%, which is attributed to the strengthening of pregnancy management since the implementation of the universal two-child policy and the improvement of the quality of obstetric practice in China in recent years. However, there was no statistically significant difference in the risk of maternal near miss for women with uterine rupture, which may be related to the increased proportions of older women, multiparous women and women with previous caesarean sections.

In this study, the lower the number of previous caesarean sections was, the higher the risk of a serious outcome in women with uterine rupture would be, which is consistent with the two studies [28, 35] but contrary to the other studies [36–38]. The outcome of uterine rupture in women without uterine scars is more serious than that of women with uterine scars, and there may be two reasons for this. First, doctors are less alert to the occurrence of uterine rupture among women without uterine scars, leading to delayed diagnosis. Second, uterine rupture among women with uterine scars often occurs in scar tissue, and there may be less bleeding [18]. There are few studies on the correlation between parity and the outcome of uterine rupture. In our study, it was believed that women with a parity of two or more were more likely have a maternal near miss or stillbirth, which was consistent with the results of the Norwegian study [38].

In terms of research design, this study could observe only the increase in the uterine rupture rate during the three birth policy periods but could not determine the causal relationship between the uterine rupture rate and the different birth policies. We believe that the increase in the proportions of pregnant women with multiple births and previous caesarean sections after the adjustment of China’s birth policy can partly explain the rise in the uterine rupture rate in recent years, but the effect of the change in birth policy on the increase in the uterine rupture rate is short-term. Studies have suggested that after the implementation of China’s universal two-child policy, the rate of caesarean sections voluntarily requested by first-time mothers may decrease due to consideration of the increased demand for reproduction and the potential harm caused by caesarean section to mothers and foetuses, and nulliparous women who wish to have a second child are more likely to choose vaginal delivery [39]. Therefore, in the long term, the universal two-child policy is conducive to reducing the caesarean section rate among nulliparous women in China, thus reducing the proportion of women with previous caesarean sections in the future and ultimately reducing the uterine rupture rate in China. However, these speculations need to be proven with rigorous data.

This study has a sample size of more than 8 million, making it one of the few studies on uterine rupture with a large sample size. The large sample size allowed us to analyse the risk of uterine rupture and the risk of maternal and foetal outcomes in women with uterine rupture among women of different ages, parity and numbers of previous caesarean sections. The quality of the data obtained by the NMNMSS is high, and the data of each monitoring hospital are entered directly into the system by trained obstetricians. Moreover, we carry out on-site quality control for medical institutions in different provinces every year to ensure the reliability of the data quality. In addition, we have provided comprehensive clarification of the uterine rupture rate in China and the changes in the incidence of uterine rupture and in maternal and foetal outcomes in women with uterine rupture during the periods of China’s different fertility policies.

However, this study still has some limitations. First, due to the limitation of the research data, we cannot distinguish between complete uterine rupture and incomplete uterine rupture. Second, due to the problems of oversampling and monitoring hospital representation, we weighted each region according to the rural and urban distribution of the 2010 census, it is not clear that this weighting fully adjusts for the oversampling of the NMNMSS in larger hospitals. Based on the above two reasons, readers should be cautious about the estimated incidence of uterine rupture in China in this study. Third, the study did not collect more detailed information, such as birth interval, whether the delivery was a TOLAC, any history of uterine surgery other than caesarean section, and the use of oxytocin or prostaglandins, which is important for an in-depth study of uterine rupture.

## Conclusions

During different birth policy periods, the uterine rupture rate in China has continued to increase, and the risk of maternal near miss among women with uterine rupture has not significantly improved. This trend requires the attention of the Chinese government, obstetricians and scholars. The Chinese government should formulate uterine rupture-related policies, management measures and disease prevention and control guidelines to reverse the high incidence of uterine rupture under the universal two-child policy period and improve maternal and foetal outcomes in women with uterine rupture. For the hospitals and obstetricians, it is necessary to conduct prudent pregnancy management and make an emergency plan for delivery of pregnant women with previous cesarean section. For scholars, more research should be carried out to verity the association between risk factors with the continuous increase of uterine rupture under different birth policies in China.

## Supplementary Information

**Additional file 1.** Number of uterine ruptures by maternal age, parity and number previous caesarean sections over different birth policy periods in 438 hospitals, China.

**Additional file 2.** Changes over different birth policy periods in age, parity and previous caesarean section distributions of women in 438 hospitals, China.

**Additional file 3.** The relative risks in different parity and previous caesatean section of women by age, China.

## Data Availability

Data in our study were collected from 438 hospitals in China’s National Maternal Near Miss Surveillance System. All data are stored electronically in an anonymous format and are currently available only to the main researchers. Data analysis collaborations may be possible on the basis of specific research proposals. Further information can be requested by e-mailing the principal investigator (liangjuan002@163.com).

## Abbreviations

WHO: World Health Organization
ACOG: American College of Obstetricians and Gynecologists
TOLAC: Trial of labor after cesarean delivery
NMNMSS: China’s National Maternal Near Miss Surveillance System
cRRs: Crude relative risks
95% CIs: 95% confidence intervals
aRRs: Adjusted relative risks
