# Supplementary material for: The incidence, risk factors and maternal and foetal outcomes of uterine rupture during different birth policy periods: an observational study in China
Source: BMC Pregnancy Childbirth. 2021 May 5;21:360. doi: 10.1186/s12884-021-03811-8 (PMC8098017; doi:10.1186/s12884-021-03811-8)
Supplement: Supplementary file 1 — Additional file 1. Number of uterine ruptures by maternal age, parity and number previous caesarean sections over different birth policy periods in 438 hospitals, China. [file 12884_2021_3811_MOESM1_ESM.pdf]

The incidence, risk factors and maternal and foetal outcomes of uterine rupture during different birth policy periods: an observational study in China

Yangwen Zhou[1], Yi Mu[2], Peiran Chen[1], Yanxia Xie[1], Jun Zhu[1,+] and Juan Liang[1,+]

Additional file 1. Number of uterine ruptures by maternal age, parity and number previous caesarean sections over different birth policy periods in 438 hospitals, China.

Maternal characteristics	One-child policy period	Partial two-child policy period	Universal two-child policy period
**Number of uterine ruptures (%)**			
**Maternal age**			
<24	242 (14.68)	344 (10.87)	519 (6.39)
24-29	588 (35.68)	1052 (33.25)	2309 (28.43)
30-34	528 (32.04)	1121 (35.43)	3181 (39.17)
≥35	233 (14.14)	553 (17.48)	2045 (25.18)
Missing	57 (3.46)	94 (2.97)	68 (0.84)
**Parity**			
0	274 (16.63)	266 (8.41)	408 (5.02)
1	1100 (66.75)	2397 (75.76)	6201 (76.35)
2	237 (14.38)	452 (14.29)	1277 (15.72)
≥3	36 (2.18)	49 (1.55)	140 (1.72)
Missing	1 (0.06)	0 (0.00)	96 (1.18)
**Previous caesarean sections**			
0	411 (24.94)	411 (12.99)	723 (8.90)
1	1090 (66.14)	2434 (76.93)	6273 (77.23)
≥2	139 (8.43)	312 (9.86)	1027 (12.64)
Missing	8 (0.49)	7 (0.22)	99 (1.22)
**Total**	1648 (100.00)	3164 (100.00)	8122 (100.00)
