# Supplementary material for: The incidence, risk factors and maternal and foetal outcomes of uterine rupture during different birth policy periods: an observational study in China
Source: BMC Pregnancy Childbirth. 2021 May 5;21:360. doi: 10.1186/s12884-021-03811-8 (PMC8098017; doi:10.1186/s12884-021-03811-8)
Supplement: Supplementary file 2 — Additional file 2. Changes over different birth policy periods in age, parity and previous caesarean section distributions of women in 438 hospitals, China. [file 12884_2021_3811_MOESM2_ESM.pdf]

Additional file 2. Changes in age, parity and previous caesarean section distributions among women in 438 hospitals over different birth policy periods in China.

Maternal characteristics	One-child policy period	Partial two-child policy period	Universal two-child policy period
**Maternal age (number of births) (%)**			
<24	883,593 (29.73)	542,423 (24.09)	536,954 (15.73)
24-29	120,0114 (40.38)	977,190 (43.39)	1,384,671 (40.56)
30-34	559,339 (18.82)	463,819 (20.59)	973,521 (28.52)
≥35	211,949 (7.13)	187,937 (8.34)	464,017 (13.59)
Missing	117,046 (3.94)	80,738 (3.58)	54,466 (1.60)
**Parity (number of births) (%)**			
0	1,905,689 (64.12)	1,287,163 (57.15)	1,647,009 (48.25)
1	918,273 (30.90)	822,020 (36.50)	1,504,703 (44.08)
2	125,139 (4.21)	121,091 (5.38)	218,916 (6.41)
≥3	21,212 (0.71)	21,476 (0.95)	35,079 (1.03)
Missing	1674 (0.06)	357 (0.02)	7922 (0.23)
**Previous caesarean sections (number of births) (%)**			
0	2,634,925 (88.66)	1,913,742 (84.98)	2,731,509 (80.02)
1	298,767 (10.05)	314,749 (13.98)	627,317 (18.38)
≥2	15,138 (0.51)	19,514 (0.87)	45,632 (1.34)
Missing	23,157 (0.78)	4102 (0.18)	9171 (0.27)
**Total**	2,971,987 (100.00)	2,252,107 (100.00)	3,413,629 (100.00)
