# Supplementary material for: The incidence, risk factors and maternal and foetal outcomes of uterine rupture during different birth policy periods: an observational study in China
Source: BMC Pregnancy Childbirth. 2021 May 5;21:360. doi: 10.1186/s12884-021-03811-8 (PMC8098017; doi:10.1186/s12884-021-03811-8)
Supplement: Supplementary file 3 — Additional file 3. The relative risks in different parity and previous caesatean section of women by age, China. [file 12884_2021_3811_MOESM3_ESM.pdf]

Additional file 3. Relative risks according to parity and number of previous caesarean sections among women in different age groups in China.

	Parity[b]					Previous caesarean sections[b]			
	**0**	**1**	**2**	**≥3**	**Missing**	**0**	**1**	**≥2**	**Missing**
**Number of uterine ruptures (%)**									
<24	191 (17.29)	765 (69.23)	141 (12.76)	6 (0.54)	2 (0.18)	236 (21.36)	763 (69.05)	101 (9.14)	5 (0.45)
24-29	395 (10.00)	2938 (74.40)	541 (13.70)	51 (1.29)	24 (0.61)	549 (13.90)	2954 (74.80)	418 (10.58)	28 (0.71)
30-34	251 (5.20)	3712 (76.85)	758 (15.69)	67 (1.39)	42 (0.87)	467 (9.67)	3738 (77.39)	574 (11.88)	51 (1.06)
≥35	79 (2.79)1	2149 (75.91)	485 (17.12)	90 (3.18)	28 (0.99)	235 (8.30)	2211 (78.10)	357 (12.61)	28 (0.99)
**Weighted rate, per 100 births[a]**									
<24	0.01	0.15	0.30	0.09	0.11	0.01	0.61	2.26	0.07
24-29	0.02	0.22	0.28	0.30	0.68	0.02	0.63	1.53	0.17
30-34	0.03	0.32	0.37	0.22	1.29	0.03	0.77	1.49	0.42
≥35	0.05	0.34	0.33	0.23	1.51	0.04	0.82	1.40	0.67
**Crude odds ratio[a]**									
<24	1	**12.37 (8.24-18.57)**	**25.18 (13.53-46.85)**	**7.99 (3.18-20.06)**	**9.17 (1.25-67.05)**	1	**51.66 (35.22-75.78)**	**192.20 (93.25-396.12)**	**6.36 (1.91-21.13)**
24-29	1	**13.31 (8.11-21.84)**	**16.88 (10.41-27.38)**	**12.30 (6.78-22.34)**	**41.49 (21.40-80.42)**	1	**39.98 (26.54-60.22)**	**96.98 (63.23-148.72)**	**10.98 (1.93-62.32)**
30-34	1	**10.23 (5.82-17.98)**	**11.87 (6.98-20.17)**	**6.94 (3.93-12.24)**	**41.12 (25.08-67.41)**	1	**26.73 (17.12-41.74)**	**51.45 (33.86-78.18)**	**14.43 (2.55-81.75)**
≥35	1	**6.76 (3.63-12.58)**	**6.51 (3.94-10.75)**	**4.48 (2.61-7.71)**	**29.76 (19.33-45.83)**	1	**23.20 (14.17-37.98)**	**39.70 (26.02-60.57)**	**18.94 (4.79-74.83)**
**Adjusted odds ratio**									
<24	1	**1.69 (1.04-2.73)**	**2.63 (1.59-4.34)**	1.20 (0.42-3.41)	-	1	**13.57 (8.36-22.03)**	**26.93 (11.64-62.28)**	2.64 (0.76-9.13)
24-29	1	**2.07 (1.13-3.78)**	**2.32 (1.25-4.31)**	**2.45 (1.25-4.81)**	3.14 (0.64-15.45)	1	**13.18 (9.09-19.11)**	**26.22 (16.28-42.23)**	1.13 (0.30-4.26)
30-34	1	**2.06 (1.16-3.66)**	**2.53 (1.38-4.62)**	**2.52 (1.28-4.59)**	3.83 (0.91-16.16)	1	**9.83 (7.08-13.64)**	**17.62 (11.22-27.65)**	1.45 (0.24-8.81)
≥35	1	1.52 (0.86-2.70)	1.69 (0.96-2.96)	**1.97 (1.03-3.78)**	5.57 (0.99-31.27)	1	**9.61 (6.72-13.76)**	**17.97 (10.52-30.72)**	0.43 (0.06-3.04)

[a]Weighted for the sampling distribution of the population covered by the Chinese National Maternal Near Miss Surveillance System. [b]We did not

adjust for parity in the different parity groups. [c]We did not adjust for previous caesarean sections in the different groups of previous caesarean sections.
